# Mortality and other adverse outcomes in patients with type 2 diabetes mellitus admitted for COVID-19 in association with glucose-lowering drugs: a nationwide cohort study

**DOI:** 10.1186/s12916-020-01832-2

**Published:** 2020-11-16

**Authors:** Luis M. Pérez-Belmonte, José David Torres-Peña, María D. López-Carmona, M. Mar. Ayala-Gutiérrez, Francisco Fuentes-Jiménez, Lucía Jorge Huerta, Jaime Alonso Muñoz, Manuel Rubio-Rivas, Manel Madrazo, Marcos Guzmán Garcia, Beatriz Vicente Montes, Joaquim Fernández Sola, Javier Ena, Ruth Gonzalez Ferrer, Carmen Mella Pérez, Carlos Jorge Ripper, Jose Javier Napal Lecumberri, Iris El Attar Acedo, Susana Plaza Canteli, Sara Fuente Cosío, Francisco Amorós Martínez, Begoña Cortés Rodríguez, Pablo Pérez-Martínez, José Manuel Ramos-Rincón, Ricardo Gómez-Huelgas, for the SEMI-COVID-19 Network

**Affiliations:** 1 Internal Medicine Department, Regional University Hospital of Málaga, Biomedical Research Institute of Málaga (IBIMA), University of Málaga (UMA), Avenida de Carlos Haya, s/n, 29010 Málaga, Spain; 2 Lipids and Atherosclerosis Unit, Department of Internal Medicine, Maimonides Biomedical Research Institute of Cordoba (IMIBIC), Reina Sofia University Hospital, University of Cordoba, Córdoba, Spain; 3 CIBER Fisiopatología de la Obesidad y Nutrición (CIBEROBN), Instituto de Salud Carlos III, Madrid, Spain; 4 Internal Medicine Department, 12 de Octubre University Hospital, Madrid, Spain; 5 Internal Medicine Department, Gregorio Marañon University Hospital, Madrid, Spain; 6 Internal Medicine Department, Bellvitge University Hospital, L’Hospitalet de Llobregat, Barcelona, Spain; 7 Internal Medicine Department, Dr. Peset University Hospital, Valencia, Spain; 8 Internal Medicine Department, San Juan de la Cruz Hospital, Úbeda (Jaén), Spain; 9 Internal Medicine Department, León University Hospital Complex, León, Spain; 10 Internal Medicine Department, Clinic Barcelona Hospital, Barcelona, Spain; 11 Internal Medicine Department, Marina Baixa Hospital, Villajoyosa (Alicante), Spain; 12 Internal Medicine Department, Tajo Hospital, Aranjuez (Madrid), Spain; 13 Internal Medicine Department, Ferrol University Hospital Complex, Ferrol (A Coruña), Spain; 14 Internal Medicine Department, Insular de Gran Canaria Hospital, Las Palmas de Gran Canaria, Spain; 15 Internal Medicine Department, Marqués de Valdecilla University Hospital, Santander, Spain; 16 Internal Medicine Department, Torrecárdenas Hospital, Almería, Spain; 17 Internal Medicine Department, Severo Ochoa University Hospital, Leganés (Madrid), Spain; 18 Internal Medicine Department, Valle del Nalón Hospital, Riaño (Langreo, Asturias), Spain; 19 Internal Medicine Department, Vinalopó University Hospital, Elche (Alicante), Spain; 20 Internal Medicine Department, Alto Guadalquivir Hospital, Andújar (Jaén), Spain; 21 Department of Clinical Medicine, Miguel Hernandez University of Elche, Alicante, Spain

**Keywords:** Type 2 diabetes mellitus, Glucose-lowering drug, Coronavirus disease 2019

## Abstract

**Background:**

Limited evidence exists on the role of glucose-lowering drugs in patients with COVID-19. Our main objective was to examine the association between in-hospital death and each routine at-home glucose-lowering drug both individually and in combination with metformin in patients with type 2 diabetes mellitus admitted for COVID-19. We also evaluated their association with the composite outcome of the need for ICU admission, invasive and non-invasive mechanical ventilation, or in-hospital death as well as on the development of in-hospital complications and a long-time hospital stay.

**Methods:**

We selected all patients with type 2 diabetes mellitus in the Spanish Society of Internal Medicine’s registry of COVID-19 patients (SEMI-COVID-19 Registry). It is an ongoing, observational, multicenter, nationwide cohort of patients admitted for COVID-19 in Spain from March 1, 2020. Each glucose-lowering drug user was matched with a user of other glucose-lowering drugs in a 1:1 manner by propensity scores. In order to assess the adequacy of propensity score matching, we used the standardized mean difference found in patient characteristics after matching. There was considered to be a significant imbalance in the group if a standardized mean difference > 10% was found. To evaluate the association between treatment and study outcomes, both conditional logit and mixed effect logistic regressions were used when the sample size was ≥ 100.

**Results:**

A total of 2666 patients were found in the SEMI-COVID-19 Registry, 1297 on glucose-lowering drugs in monotherapy and 465 in combination with metformin. After propensity matching, 249 patients on metformin, 105 on dipeptidyl peptidase-4 inhibitors, 129 on insulin, 127 on metformin/dipeptidyl peptidase-4 inhibitors, 34 on metformin/sodium-glucose cotransporter 2 inhibitor, and 67 on metformin/insulin were selected. No at-home glucose-lowering drugs showed a significant association with in-hospital death; the composite outcome of the need of intensive care unit admission, mechanical ventilation, or in-hospital death; in-hospital complications; or long-time hospital stays.

**Conclusions:**

In patients with type 2 diabetes mellitus admitted for COVID-19, at-home glucose-lowering drugs showed no significant association with mortality and adverse outcomes. Given the close relationship between diabetes and COVID-19 and the limited evidence on the role of glucose-lowering drugs, prospective studies are needed.

**Supplementary information:**

**Supplementary information** accompanies this paper at 10.1186/s12916-020-01832-2.

## Background

The novel coronavirus disease 2019 (COVID-19) caused by the severe acute respiratory syndrome coronavirus 2 (SARS-CoV-2) initially emerged in China in December 2019 and quickly spread around the world, causing a global pandemic [1].

For patients with COVID-19, diabetes has been reported as one of the most frequent comorbidities, occurring in around 20% of patients [2, 3]. It is well known that type 2 diabetes mellitus (T2DM) may negatively impact clinical outcomes in patients with COVID-19. Possible poor outcomes include moderate and severe cases of COVID-19 disease, a higher rate of hospitalized patients in intensive care unit (ICU), a higher rate of treatment with anti-interleukin 6 receptor antibody (tocilizumab), and higher mortality [4]. Furthermore, it has been reported that hyperglycemia during COVID-19 infection, and particularly at hospital admission, has been associated with worse COVID-19 outcomes and could be a prognostic factor for worse outcomes in patients with or without T2DM [5, 6]. Indeed, patients with hyperglycemia may experience reduced effect of anti-COVID-19 therapies, particularly tocilizumab, an anti-interleukin 6 receptor antibody indicated for patients with moderate-to-severe COVID-19 pneumonia [7]. Thus, not only T2DM status but also the presence of hyperglycemia could have unfavorable effects on hospital admission, clinical outcomes, and drug therapy, leading to a worse prognosis in COVID-19 patients [5, 6].

However, to date, there is no conclusive evidence in regard to the potential implications on adverse outcomes of glucose-lowering drugs in patients with COVID-19 [8–11]. In a recent study, the use of oral glucose-lowering medications (metformin, α-glucosidase, secretagogues, and dipeptidyl peptidase-4 inhibitors (DPP-4i)) showed neutral effects on in-hospital mortality and the composite outcome of poor prognosis defined as progression to severe or critical illness and in-hospital death. In contrast, insulin usage was associated with a greater risk of poor prognosis compared to not using it [12]. Other studies have reported a beneficial effect of metformin and DPP-4i on clinical outcomes in patients hospitalized with COVID-19 [13, 14]. The potential effects of these medications on COVID-19-related pathological mechanisms, such as systemic inflammation and endothelial dysfunction, have been proposed as the underlying mechanisms of possible benefits in reports on mechanistic hypotheses and preliminary data [10, 15, 16].

We conducted this study based on the premise that patients with T2DM seem particularly prone to a worse prognosis if infected by SARS-CoV-2, and in response to the fact that there is only controversial, limited evidence on the role of glucose-lowering drugs on clinical adverse outcomes. Our main objective was to examine the association between in-hospital death and each routine at-home glucose-lowering drug both individually and in combination with metformin in patients with T2DM admitted for COVID-19. We also evaluated their association with the composite outcome of need for ICU admission, invasive and non-invasive mechanical ventilation, or in-hospital death as well as on the development of in-hospital complications and a long-time hospital stay.

## Methods

### Study design and population

We selected all patients with T2DM included in the Spanish Society of Internal Medicine’s registry of COVID-19 patients (SEMI-COVID-19 Registry) [17] from March 1, 2020, to the study’s cutoff date of July 19, 2020. The SEMI-COVID-19 Registry is an ongoing, observational, multicenter, nationwide cohort of patients admitted for COVID-19 in Spain from March 1, 2020, whose main objective is to obtain detailed information on the epidemiology, clinical progress, and treatment received by patients with COVID-19 in real-world clinical practice at admission and during hospitalization. It retrospectively compiles sociodemographic variables, previous medical history, routine at-home treatments, clinical presentation, the patient’s clinical condition (including the degree of functional dependence as evaluated by the Barthel Index [18] and the presence of comorbidities as evaluated by the Charlson Comorbidity Index [19]), laboratory test results (blood gas analysis, complete blood count, coagulation tests, glucose, creatinine, urea, lactate dehydrogenase, alanine aminotransferase, aspartate aminotransferase, alkaline phosphatase, bilirubin, sodium, potassium, triglycerides, creatine kinase, ferritin, lactic acid, c-reactive protein, procalcitonin, interleukin 6, d-dimer, troponin, albumin), radiological findings (chest X-ray, chest-computerized tomography scan, lung ultrasound), clinical management, hospital complications, hospital stay, and in-hospital death from the first admission for COVID-19 of patients who are aged 18 years of age or older.

An online electronic data capture system was developed for the registry, including a database manager along with procedures for the verification of data and contrasting of information against the original medical record in order to ensure the best possible quality of data collection. The database platform is hosted on a secure server. All information contained in the database, the configuration of the information within the database, and the database itself are fully encrypted. Every client-server data transfer is encrypted through a valid TLS certificate. Daily backups are performed in order to ensure data integrity.

A diagnosis of T2DM was ascertained either through a T2DM diagnosis in medical records or a self-reported diagnosis confirmed by medical records reviewed by physicians. T2DM was defined according to the most recent American Diabetes Association guidelines [20].

In-hospital complications include the onset of at least one of the following: secondary bacterial pneumonia, acute respiratory distress syndrome, acute heart failure, arrhythmia, acute coronary syndrome, myocarditis, epileptic seizures, stroke, shock, sepsis, acute kidney failure, disseminated intravascular coagulation, venous thromboembolism, multiple organ dysfunction syndrome, acute limb ischemia, ICU admission, and need for ventilation support, including invasive and non-invasive mechanical ventilation or high-flow oxygen therapy. A long hospital stay was defined as hospitalization longer than the median length of stay in days for all patients included in this study.

### Diagnosis and severity grade of COVID-19 disease

The presence of COVID-19 in respiratory specimens was established by RNA detection of SARS-CoV-2 using real-time reverse transcription-polymerase chain reaction methods as indicated by the international literature on COVID-19 infection [1]. Diagnostic testing for SARS-CoV-2 was performed in appropriately equipped laboratories by staff trained in the relevant technical and safety procedures. The COVID-19 pneumonia severity grade was established according to the patient’s clinical condition: mild grade (symptoms without evidence of pneumonia or hypoxia), moderate grade (clinical signs of pneumonia but no signs of severe pneumonia, including basal oxygen saturation ≥ 92%), severe grade (clinical signs of pneumonia plus one of the following: basal oxygen saturation < 92%, resting respiratory rate > 30 breaths/min, severe respiratory distress), and critical grade (sepsis or shock with acute respiratory distress syndrome and/or multiple organ dysfunction or failure).

### Study outcomes

The primary outcome was in-hospital death according to each at-home glucose-lowering drug in monotherapy or in dual therapy with metformin. Secondary outcomes were the following: first, a composite outcome including the need for ICU admission, invasive and non-invasive mechanical ventilation, or in-hospital death; second, in-hospital complications; and third, a long hospital stay.

### Statistical analysis

The characteristics of patients with T2DM included in the registry were analyzed using descriptive statistics. Continuous and categorical variables were expressed as means ± standard deviation and as absolute value and percentage, respectively. The differences between the groups were determined using the two-sample Student’s *t* test or the Mann-Whitney-Wilcoxon rank-sum test for continuous variables and Pearson’s chi-squared test for categorical variables. Values were considered to be statistically significant when *p* < 0.05.

We grouped patients according to glucose-lowering drugs in monotherapy and in combination with metformin. In order to match each patient in one of these groups with a patient of another group receiving other glucose-lowering drugs in a 1:1 manner, propensity scores using nearest neighbor matching with a caliper of 0.1 and a greedy matching algorithm were used. The probability of a patient being treated with one glucose-lowering drug as opposed to other glucose-lowering drugs was estimated using a logistic regression model that included variables that could have affected the outcomes as independent variables (age; gender; history of smoking, hypertension; dyslipidemia; chronic kidney disease; cerebrovascular disease; chronic obstructive pulmonary disease; atrial fibrillation; coronary artery disease; heart failure; obesity; dementia; Barthel Index score; and Charlson Comorbidity Index score; treatment with angiotensin-converting enzyme inhibitor, angiotensin II receptor blocker, anticoagulant, and statin; admission blood glucose; serum creatinine; and transaminase levels). In order to assess the adequacy of propensity matching, we used the standardized mean difference found in patient characteristics after matching. There was considered to be a significant imbalance in the group if a standardized mean difference between baseline variables of greater than 10% was found.

According to the findings of Austin [21], the statistical models for paired samples are preferable to models for the analysis of independent samples. For this reason, in order to evaluate the association between treatment and study outcomes, both conditional logit and mixed effect (matched pairs as random effects) logistic regressions were used. In case the sample size was smaller than 100, the McNemar test for matched data was performed to evaluate the association. We also performed univariate and multivariate logistic regression models adjusted with confounding variables in order to estimate the treatment effect using the totality of the data, as a sensitivity analysis. Statistical analyses were performed using the R software, version 3.6.2.

## Results

### Baseline clinical variables and treatments

A total of 2666 patients with T2DM admitted for COVID-19 were included in this study. Baseline sociodemographic, clinical, and therapeutic variables are shown in Table 1. In regard to glucose-lowering drugs, metformin was the most frequently used (60.8%), followed by DPP-4i (30.2%), insulin (27.6%), sodium-glucose cotransporter 2 inhibitor (SGLT-2i) (11.3%), and glucagon-like peptide-1 receptor agonist (GLP-1RA) (4.8%).

**Table 1:** Baseline sociodemographic clinical and therapeutic characteristics of patients with type 2 diabetes mellitus hospitalized for coronavirus disease 2019

Variables	*n* = 2666
Age (years)	74.9 ± 8.4
Male gender	1647 (61.9%)
Obesity	766 (31.6%)
Admission blood glucose (mg/dL)	153 ± 45.7
Admission serum creatinine (mg/dL)	1.03 ± 0.23
Admission aspartate aminotransferase (U/L)	31 ± 8
Admission alanine aminotransferase (U/L)	26 ± 6
Metformin	1618 (60.8%)
DPP-4i	791 (30.2%)
GLP1-1RA	127 (4.8%)
SGLT-2i	296 (11.3%)
Insulin	723 (27.6%)
Angiotensin-converting enzyme inhibitor	724 (27.4%)
Angiotensin II receptor blocker	789 (29.9%)
Statin	1534 (58.0%)
Antiplatelet	643 (24.8%)
Anticoagulant	(16.3%)
History of smoking	955 (35.8%)
Hypertension	2026 (76.2%)
Dyslipidemia	1730 (65.0%)
Chronic kidney disease	352 (13.2%)
Atrial fibrillation	444 (16.7%)
Coronary artery disease	610 (22.9%)
Heart failure	445 (16.7%)
Chronic obstructive pulmonary disease	346 (13.0%)
Liver disease	172 (6.5%)
Cancer	353 (13.3%)
Stroke	322 (12.1%)
Dementia	384 (14.4%)
Depression	308 (11.6%)
Moderate-severe functional dependence	636 (24.2%)
Moderate-severe comorbidity	2338 (90.7%)
Disease severity	
Moderate	1891 (70.9%)
Severe	714 (26.8%)
Critical	61 (2.3%)
Hydroxychloroquine	2185 (82.0%)
Chloroquine	72 (2.7%)
Lopinavir/ritonavir	1195 (44.8%)
Azithromycin	1595 (59.8%)
Remdesivir	36 (1.4%)
Interferon-β	276 (10.4%)
Corticosteroids	1186 (44.5%)
Tocilizumab	215 (8.1%)

Values are shown as mean ± standard deviations, absolute values, and percentages

The degree of functional dependence was assessed using the Barthel Index. The presence of comorbidities was assessed using the Charlson Comorbidity Index

*DPP-4i* dipeptidyl peptidase-4 inhibitors, *GLP-1RA* glucagon-like peptide-1 receptor agonist, *mg/dL* milligram/deciliter, *SGLT-2i* sodium-glucose cotransporter 2 inhibitor, *U/L* unit/liter

**Fig. 1:**
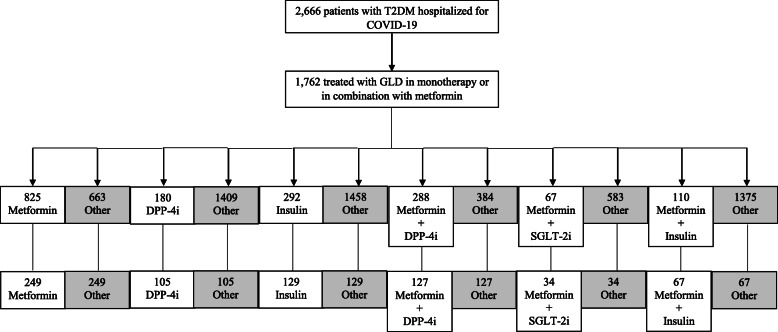
Flow chart for pre- and post-propensity score matching indicating the number of patients in each compared group. COVID-19, coronavirus disease 2019; DPP-4i, dipeptidyl peptidase-4 inhibitors; GLD, glucose-lowering drugs; GLP-1RA, glucagon-like peptide-1 receptor agonists; SGLT-2i, sodium-glucose transporter 2 inhibitors; T2DM, type 2 diabetes mellitus

Of the total number of patients, 1297 (48.6%) were treated with glucose-lowering drugs in monotherapy (825 with metformin (63.6%), 180 with DPP-4i (13.9%), and 292 with insulin (22.5%)). Dual therapy of metformin in combination with other glucose-lowering drugs was used in 465 patients (17.4%) (288 with metformin plus DPP-4i (61.9%), 67 metformin plus SGLT-2i (14.4%), and 110 metformin plus insulin (23.7%)). Following 1:1 propensity score matching of each glucose-lowering drug alone or in combination with metformin versus the other glucose-lowering drugs, 249 patients were included in the metformin group, 105 in the DPP-4i group, 129 in the insulin group, 127 in the metformin plus DPP-4i group, 34 in the metformin plus SGLT-2i group, and 67 in the metformin plus insulin group. No patients were identified with GLP-1RA alone, SGLT-2i alone, or metformin plus GLP-1RA. A flow chart for pre- and post-propensity score matching showing patients in each compared group can be found in Fig. 1.

### Pre- and post-propensity matching characteristics

The pre- and post-propensity score matching of the baseline sociodemographic and clinical characteristics of each glucose-lowering drug alone or in combination with metformin versus the other glucose-lowering drugs used by patients before hospitalization for COVID-19 are shown in Additional file 1: Table S1; Additional file 2: Table S2; Additional file 3: Table S3; Additional file 4: Table S4; Additional file 5: Table S5; and Additional file 6: Table S6. After propensity matching, the groups were well-balanced, and only negligible differences were observed (standardized mean difference ≤ 0.1).

### Associations between treatments and study outcomes

After propensity score matching, no differences were found in in-hospital deaths; the composite outcome of need for ICU admission, invasive and non-invasive mechanical ventilation, or in-hospital death; in-hospital complications; and a long-time hospital stay according to the glucose-lowering drug used. None of the at-home glucose-lowering drugs analyzed showed significant association with study outcomes after applying both the conditional logit and mixed effect logistic regression if the sample size was ≥ 100 or McNemar test if the sample size was < 100. The association between each glucose-lowering drug group and the study outcomes after propensity matching is shown in Tables 2 and 3.

**Table 2:** Association between metformin, dipeptidyl peptidase-4 inhibitors, insulin, and metformin plus dipeptidyl peptidase-4 inhibitors and study outcomes after propensity score matching

Outcomes	Treatment groups			Conditional logit logistic regression		Mixed effect logistic regression	
				OR (95% CI)	*p* value	OR (95% CI)	*p* value
	Metformin (*n* = 249)	Other GLD (*n* = 249)	*p* value				
In-hospital deaths	79 (31.7%)	79 (31.7%)	1.000	1.15 (0.78–1.71)	0.482	1.16 (0.78–1.72)	0.482
ICU admission, mechanical ventilation, or in-hospital death	91 (36.5%)	88 (35.4%)	0.852	1.05 (0.73–1.51)	0.780	1.05 (0.73–1.52)	0.779
In-hospital complications	146 (58.6%)	139 (55.8%)	0.587	1.18 (0.81–1.72)	0.392	1.17 (0.81–1.70)	0.398
Long-time hospital stay	59 (23.7%)	45 (18.1%)	0.152	1.49 (0.95–2.31)	0.079	1.49 (0.96–2.33)	0.075
	DPP-4i (*n* = 105)	Other GLD (*n* = 105)	*p* value				
In-hospital deaths	41 (39.0%)	44 (41.9%)	0.779	1.05 (0.66–2.13)	0.521	1.05 (0.67–2.11)	0.562
ICU admission, mechanical ventilation, or in-hospital death	45 (42.9%)	42 (40.0%)	0.780	1.12 (0.65–1.92)	0.680	1.12 (0.65–1.95)	0.675
In-hospital complications	66 (62.9%)	70 (66.7%)	0.665	0.94 (0.67–2.12)	0.480	0.94 (0.67–2.13)	0.481
Long-time hospital stay	23 (21.9%)	24 (22.9%)	1.000	0.83 (0.38–1.46)	0.400	0.84 (0.39–1.46)	0.406
	Insulin (*n* = 129)	Other GLD (*n* = 129)	*p* value				
In-hospital deaths	51 (39.5%)	46 (35.7%)	0.607	1.15 (0.65–1.97)	0.590	1.15 (0.65–1.97)	0.598
ICU admission, mechanical ventilation, or in-hospital death	57 (44.2%)	54 (41.9%)	0.802	1.10 (0.67–1.80)	0.710	1.10 (0.67–1.81)	0.706
In-hospital complications	49 (38.9%)	46 (35.7%)	0.796	1.00 (0.58–1.67)	0.930	1.00 (0.57–1.67)	0.912
Long-time hospital stay	22 (17.1%)	34 (26.4%)	0.097	0.63 (0.37–1.20)	0.158	0.63 (0.37–1.21)	0.159
	Metformin + DPP-4i (*n* = 127)	Other GLD (*n* = 127)	*p* value				
In-hospital deaths	29 (22.8%)	37 (29.1%)	0.317	0.73 (0.40–1.28)	0.270	0.72 (0.39–1.27)	0.251
ICU admission, mechanical ventilation, or in-hospital death	40 (31.5%)	45 (35.4%)	0.592	0.84 (0.50–1.41)	0.510	0.84 (0.49–1.41)	0.503
In-hospital complications	67 (52.8%)	70 (55.1%)	0.801	0.87 (0.51–1.46)	0.596	0.86 (0.50–1.47)	0.592
Long-time hospital stay	29 (22.8%)	26 (20.5%)	0.761	1.17 (0.62–2.23)	0.627	1.17 (0.62–2.19)	0.632

Data are shown as absolute values and percentages. A significant imbalance in the group was considered if a standardized mean difference between baseline variables of greater than 10% was found. Values were considered to be statistically significant when *p* < 0.05

*DPP-4i* dipeptidyl peptidase-4 inhibitors, *GLD* glucose-lowering drugs, *ICU* intensive care unit, *OR* odds ratio, *95% CI* 95% confidence interval

**Table 3:** Association between metformin plus sodium-glucose transporter 2 inhibitors and metformin plus insulin and study outcomes after propensity score matching

Outcomes	Treatment groups			McNemar test
	Metformin + SGLT-2i (*n* = 34)	Other GLD (n = 34)	*p* value	
In-hospital deaths	6 (17.6%)	10 (29.4%)	0.391	1.000
ICU admission, mechanical ventilation, or in-hospital death	10 (29.4%)	11 (32.4%)	0.987	0.965
In-hospital complications	19 (55.9%)	22 (64.7%)	0.620	0.201
Long-time hospital stay	6 (17.6%)	4 (11.8%)	0.732	0.838
	Metformin + insulin (*n* = 67)	Other GLD (*n* = 67)	*p* value	
In-hospital deaths	24 (35.8%)	23 (34.3%)	1.000	0.521
ICU admission, mechanical ventilation, or in-hospital death	46 (68.7%)	36 (53.7%)	0.112	0.111
In-hospital complications	44 (65.7%)	36 (53.7%)	0.218	0.480
Long-time hospital stay	15 (22.4%)	13 (19.4%)	0.832	0.870

Data are shown as absolute values and percentages. Values were considered to be statistically significant when *p* < 0.05

*GLD* glucose-lowering drugs, *ICU* intensive care unit, *SGLT-2i* sodium-glucose transporter 2 inhibitors

Before matching, patients treated at home with metformin in monotherapy, compared to patients who were treated with other glucose-lowering drugs, had a lower rate of in-hospital deaths (29.6% vs 36.8%, *p* = 0.005); the composite outcome of ICU admission, mechanical ventilation, or in-hospital death (35.8% vs 41.9%, *p* = 0.023); and in-hospital complications (52.7% vs 59.4%, *p* = 0.013). A lower in-hospital death rate was also found in patients with metformin plus DPP-4i (27.8% vs 35.4%, *p* = 0.044) and in patients with metformin plus SGLT-2i (19.4% vs 39.1%, *p* = 0.003). On the other hand, a higher rate of in-hospital deaths was observed among patients with DPP-4i alone and insulin alone (41.7% vs 31.2%, *p* = 0.007; 38% vs 29.6%, *p* = 0.007, respectively). Patients with insulin also had a higher rate of the composite outcome of the need for ICU admission, mechanical ventilation, or in-hospital death (42.8% vs 36.2%, *p* = 0.039). Patients treated with metformin and insulin were found to have higher rates of in-hospital complications (66.4% vs 55.0%, *p* = 0.030).

On the univariate models, metformin was significantly associated with a lower rate of in-hospital deaths (odds ratio (OR) 0.73, 95% confidence interval (CI) 0.58–0.90, *p* = 0.004); the need for ICU admission, mechanical ventilation, or in-hospital death (OR 0.78, 95% CI 0.63–0.96, *p* = 0.020); and in-hospital complications (OR 0.76, 95% CI 0.62–0.94, *p* = 0.011). Metformin in combination with DPP-4i was associated with a lower in-hospital death rate (OR 0.70, 95% CI 0.50–0.98, *p* = 0.036). Otherwise, DPP-4i and insulin were associated with higher in-hospital deaths (OR 1.56, 95% CI 1.13–2.14, *p* = 0.006; OR 1.45, 95% CI 1.11–1.88, *p* = 0.006; respectively). The composite outcome of the need for ICU admission, mechanical ventilation, or in-hospital death was also higher in patients on insulin (OR 1.32, 95% CI 1.02–1.71, *p* = 0.033). These associations were not found when models were fully adjusted using multivariate logistic regression. The association between each glucose-lowering drug group and the study outcomes before propensity score matching is shown in Tables 4 and 5.

**Table 4:** Association between metformin, dipeptidyl peptidase-4 inhibitors, insulin, and metformin plus dipeptidyl peptidase-4 inhibitors and study outcomes before propensity score matching

Outcomes	Treatment groups			Univariate model		Multivariate model	
				OR (95% CI)	*p* value	OR (95% CI)	*p* value
	Metformin (*n* = 825)	Other GLD (*n* = 663)	*p* value				
In-hospital deaths	244 (29.6%)	244 (36.8%)	0.005	0.73 (0.58–0.90)	0.004	1.10 (0.76–1.60)	0.616
ICU admission, mechanical ventilation, or in-hospital death	295 (35.8%)	278 (41.9%)	0.023	0.78 (0.63–0.96)	0.020	1.03 (0.73–1.44)	0.883
In-hospital complications	435 (52.7%)	394 (59.4%)	0.013	0.76 (0.62–0.94)	0.011	1.08 (0.77–1.50)	0.666
Long-time hospital stay	195 (23.6%)	149 (22.5%)	0.584	1.08 (0.85–1.38)	0.542	1.41 (0.96–2.09)	0.080
	DPP-4i (*n* = 180)	Other GLD (*n* = 1409)	*p* value				
In-hospital deaths	75 (41.7%)	440 (31.2%)	0.007	1.56 (1.13–2.14)	0.006	1.39 (0.64–1.67)	0.876
ICU admission, mechanical ventilation, or in-hospital death	80 (44.4%)	531 (37.7%)	0.119	1.30 (0.95–1.78)	0.102	0.97 (0.61–1.52)	0.890
In-hospital complications	113 (62.8%)	775 (55.0%)	0.104	1.33 (0.96–1.84)	0.089	0.95 (0.59–1.54)	0.842
Long-time hospital stay	35 (19.4%)	321 (22.8%)	0.333	0.81 (0.54–1.18)	0.288	0.90 (0.52–1.52)	0.703
	Insulin (*n* = 292)	Other GLD (*n* = 1458)	*p* value				
In-hospital deaths	111 (38.0%)	431 (29.6%)	0.007	1.45 (1.11–1.88)	0.006	1.11 (0.70–1.75)	0.652
ICU admission, mechanical ventilation, or in-hospital death	125 (42.8%)	528 (36.2%)	0.039	1.32 (1.02–1.71)	0.033	1.09 (0.71–1.66)	0.699
In-hospital complications	170 (58.2%)	786 (53.9%)	0.143	1.23 (0.95–1.60)	0.126	0.96 (0.62–1.47)	0.834
Long-time hospital stay	74 (25.3%)	327 (22.4%)	0.357	1.16 (0.86–1.55)	0.318	0.699 (0.43–1.12)	0.145
	Metformin + DPP-4i (*n* = 288)	Other GLD (*n* = 384)	*p* value				
In-hospital deaths	80 (27.8%)	136 (35.4%)	0.044	0.70 (0.50–0.98)	0.036	0.71 (0.40–1.28)	0.257
ICU admission, mechanical ventilation, or in-hospital death	104 (36.1%)	159 (41.4%)	0.159	0.79 (0.57–1.08)	0.137	0.82 (0.50–1.36)	0.452
In-hospital complications	160 (55.6%)	226 (58.9%)	0.304	0.84 (0.61–1.15)	0.268	1.01 (0.63–1.62)	0.975
Long-time hospital stay	71 (24.7%)	95 (24.7%)	1.000	1.00 (0.71–1.44)	0.963	1.42 (0.82–2.47)	0.217

Data are shown as absolute values and percentages. Values were considered to be statistically significant when *p* < 0.05

*DPP-4i* dipeptidyl peptidase-4 inhibitors, *GLD* glucose-lowering drugs, *ICU* intensive care unit, *OR* odds ratio, *95% CI* 95% confidence interval

**Table 5:** Association between metformin plus sodium-glucose transporter 2 inhibitors and metformin plus insulin and study outcomes before propensity score matching

Outcomes	Treatment groups		
	Metformin + SGLT-2i (*n* = 67)	Other GLD (*n* = 583)	*p* value
In-hospital deaths	13 (19.4%)	228 (39.1%)	0.003
ICU admission, mechanical ventilation, or in-hospital death	35 (52.2%)	375 (64.3%)	0.051
In-hospital complications	33 (49.3%)	351 (60.2%)	0.132
Long-time hospital stay	8 (11.9%)	130 (22.3%)	0.087
	Metformin + insulin (*n* = 110)	Other GLD (*n* = 1375)	*p* value
In-hospital deaths	43 (39.1%)	445 (32.4%)	0.175
ICU admission, mechanical ventilation, or in-hospital death	76 (69.1%)	810 (58.9%)	0.084
In-hospital complications	73 (66.4%)	756 (55.0%)	0.030
Long-time hospital stay	21 (19.1%)	320 (23.3%)	0.360

Data are shown as absolute values and percentages. Values were considered to be statistically significant when *p* < 0.05

*GLD* glucose-lowering drugs, *ICU* intensive care unit, *SGLT-2i* sodium-glucose transporter 2 inhibitors

## Discussion

Our study found that none of the at-home glucose-lowering drugs analyzed (metformin, DPP-4i, insulin, metformin plus DPP-4i, metformin plus SGLT-2i, and metformin plus insulin) showed a significant association with in-hospital deaths; the composite outcome of the need for ICU, mechanical ventilation, or in-hospital death; in-hospital complications; or a long-time hospital stay.

Currently, only limited evidence is available on the role of glucose-lowering drugs in adverse clinical outcomes. Recently, a study conducted by Chen et al. [12] focused on the impact of glucose-lowering medications on the clinical outcomes of patients with diabetes and COVID-19, showing that insulin users had a greater risk of a composite outcome of progression to severe or critical illness and in-hospital death compared with non-insulin users (OR 3.58, 95% CI 1.37–9.35, *p* = 0.009). However, none of the glucose-lowering medications (metformin, DPP-4i, α-glucosidase, secretagogues, and insulin) was associated with in-hospital deaths. In our study, all glucose-lowering medications evaluated, including oral drugs and insulin, had no significant association with in-hospital adverse outcomes in patients admitted for COVID-19. The differences in the definitions of the outcomes of clinical complications during the hospitalization, the methodology used, and the sample size could explain the difference found regarding the role of insulin. We included an important number of patients taking glucose-lowering drugs and performed a propensity score matching that included variables that could affect the treatment choice or adverse outcomes as independent variables.

Two observational studies have specifically evaluated the role of metformin and DPP-4i in patients admitted for COVID-19 [13, 14]. The study focused on metformin, conducted by Bramante et al. [13] in the USA, found no significant reduction in in-hospital mortality in the overall sample, which is in line with our results. However, when a subgroup analysis was performed, women with obesity and T2DM who used metformin were observed to have a significantly lower mortality rate. Metformin has been proposed to reduce levels of IL-10, IL-6, and TNFα—important mediators in macrophage activation and cytokine release; improves neutrophil/lymphocyte ratio; stabilizes mast cells; decreases thrombosis; and improves endothelial function, thus reducing the adverse impacts of mortality and complication in patients with COVID-19 [13, 22–27]. In the study of Rhee et al. [14] from Korea, protective effects against severe/lethal cases were shown in patients with T2DM who used DPP-4i in monotherapy or in combination with renin-angiotensin system blockers after adjusting for age, gender, comorbidity, and medications [14]. It is known that DPP-4i have anti-fibrotic activity and modulate inflammation and that these properties could be helpful in reducing the progression towards a hyperinflammatory state associated with severe COVID-19 [14, 28, 29]. All these major inflammatory mechanisms observed in some COVID-19 patients suggest that the endothelium could be a key target organ in COVID-19 infection [30]. The role of renin-angiotensin system blockers in hypertensive patients with COVID-19 has also been widely discussed [31]. Although hypertension has been associated with a higher risk of mortality, recent studies have shown that previous treatment with angiotensin-converting enzyme inhibitors and angiotensin II receptor blockers did not alter the outcomes in hypertensive patients [31, 32]. In our study, no significant associations with adverse clinical outcomes were found among users of DPP-4i and users of the other glucose-lowering drugs. This difference could be explained by the different methodology used in our study, which includes an important adjustment using a propensity score matching in order to compare the two groups of glucose-lowering drugs, as well as the differences in the baseline characteristics, with older patients and higher comorbidity found in our study compared with Rhee et al.’s study (mean age of 74.9 ± 8.4 vs 63.7 ± 12.2 years old).

Other glucose-lowering treatments in our study, such as metformin in combination with SGLT-2i or with insulin, showed no significant differences compared with the other glucose-lowering treatments. No propensity score matching was performed for these two comparisons due to the small number of patients in these treatment groups, so no solid conclusions may be drawn. To our knowledge, no evidence exists about the role of SGLT-2i on adverse outcomes in patients with COVID-19. A hypothetical anti-viral effect of SGLT-2i has been suggested, as these agents can increase lactate concentrations and decrease intracellular pH, which could reduce the viral load [33].

Our findings are important because they provide valuable information on the role of at-home glucose-lowering drugs on adverse outcomes in patients with T2DM admitted for COVID-19. In addition, this is the first study to report the role of each glucose-lowering drugs alone or in combination with metformin compared to the other glucose-lowering drugs after a robust adjustment using a wide number of confounding variables. Furthermore, data were collected in a large multicenter, nationwide study. Nevertheless, these results should be considered within the context of several potential limitations. Despite the propensity matching analysis performed and due to the fact that the data were obtained retrospectively via medical records from the electronic medical record system, the possible effects of unmeasured confounding factors cannot be excluded. In addition, some glucose-lowering drug group comparisons had a small number of patients after propensity score matching that could be an explanation for the lack of significant differences. No other intermediate adverse outcomes were explored due to their reduced number. Furthermore, we did not record the characteristics of T2DM, such as glycemic control before hospitalization, duration of diabetes, blood glucose levels during the hospitalization, or in-hospital anti-hyperglycemic management. Lastly, the data provided about at-home glucose-lowering drugs did not include information on treatment adherence or treatment duration.

## Conclusions

Our study found that the at-home use of metformin, DPP-4i, insulin, metformin plus DPP-4i, metformin plus SGLT-2i, and metformin plus insulin showed no significant association with in-hospital deaths; the composite outcome of need for ICU admission, mechanical ventilation, or in-hospital death; in-hospital complications; or long-time hospital stay in patients with T2DM admitted for COVID-19. Given the large number of patients with T2DM and COVID-19 infection, the ominous relationship between these pathologies, and the limited evidence on the role of glucose-lowering drugs, prospective studies are needed.

## Supplementary Information

**Additional file 1:** **Table S1.** Pre- and post-propensity score matching of baseline sociodemographic and clinical characteristics of patients with type 2 diabetes mellitus admitted for coronavirus disease 2019 treated with metformin versus other glucose-lowering drugs.

**Additional file 2:** **Table S2.** Pre- and post-propensity score matching of baseline sociodemographic and clinical characteristics of patients with type 2 diabetes mellitus admitted for coronavirus disease 2019 treated with dipeptidyl peptidase-4 inhibitors versus other glucose-lowering drugs.

**Additional file 3:** **Table S3.** Pre- and post-propensity score matching of baseline sociodemographic and clinical characteristics of patients with type 2 diabetes mellitus admitted for coronavirus disease 2019 treated with insulin versus other glucose-lowering drugs.

**Additional file 4:** **Table S4.** Pre- and post-propensity score matching of baseline sociodemographic and clinical characteristics of patients with type 2 diabetes mellitus admitted for coronavirus disease 2019 treated with metformin plus dipeptidyl peptidase-4 inhibitors versus other glucose-lowering drugs.

**Additional file 5:** **Table S5.** Pre- and post-propensity score matching of baseline sociodemographic and clinical characteristics of patients with type 2 diabetes mellitus admitted for coronavirus disease 2019 treated with metformin plus sodium-glucose transporter 2 inhibitors versus other glucose-lowering drugs.

**Additional file 6:** **Table S6.** Pre- and post-propensity score matching of baseline sociodemographic and clinical characteristics of patients with type 2 diabetes mellitus admitted for coronavirus disease 2019 treated with metformin plus insulin versus other glucose-lowering drugs.

**Additional file 7.**

## Data Availability

All data generated or analyzed during this study are included in this published article and its supplementary information files.

## Abbreviations

CI: Confidence interval
COVID-19: Coronavirus disease 2019
DPP-4i: Dipeptidyl peptidase-4 inhibitors
GLP-1RA: Glucagon-like peptide-1 receptor agonist
ICU: Intensive care unit
OR: Odds ratio
SARS-Cov-2: Severe acute respiratory syndrome coronavirus 2
SGLT-2i: Sodium-glucose cotransporter 2 inhibitor
T2DM: Type 2 diabetes mellitus
