# Supplementary material for: Mortality and other adverse outcomes in patients with type 2 diabetes mellitus admitted for COVID-19 in association with glucose-lowering drugs: a nationwide cohort study
Source: BMC Med. 2020 Nov 16;18:359. doi: 10.1186/s12916-020-01832-2 (PMC7666969; doi:10.1186/s12916-020-01832-2)
Supplement: Supplementary file 1 — Additional file 1: Table S1. Pre- and post-propensity score matching of baseline sociodemographic and clinical characteristics of patients with type 2 diabetes mellitus admitted for coronavirus disease 2019 treated with metformin versus other glucose-lowering drugs. [file 12916_2020_1832_MOESM1_ESM.docx]

Additional file 1: Table S1. Pre- and post-propensity score matching of baseline sociodemographic and clinical characteristics of patients with type 2 diabetes mellitus admitted for coronavirus disease 2019 treated with metformin versus other glucose-lowering drugs.

|  | Pre-propensity matching | | | | Post-propensity matching | | | |
| --- | --- | --- | --- | --- | --- | --- | --- | --- |
|  | Metformin (n=825) | Other GLD (n=663) | p-value | SMD | Metformin (n=249) | Other GLD (n=249) | p-value | SMD |
| Age (years) | 74.8 ± 7.9 | 77.1 ± 7.1 | 0.002 | 0.112 | 73.4 ± 6.8 | 75.8 ± 7.0 | 0.336 | 0.013 |
| Male gender | 542 (65.7%) | 379 (57.2%) | 0.005 | 0.179 | 153 (61.4%) | 145 (58.2%) | 0.522 | 0.066 |
| Body Mass Index ≥30 | 218 (26.4%) | 173 (26.1%) | 0.788 | 0.018 | 77 (30.9%) | 70 (28.1%) | 0.556 | 0.062 |
| Admission BG (mg/dL) | 144.0 ± 22.4 | 168.0 ± 35.2 | <0.001 | 0.352 | 155.5 ± 33.2 | 163.2 ± 34.8 | 0.264 | 0.099 |
| Admission serum creatinine (md/dL) | 0.96 ± 0.20 | 1.35 ± 0.51 | <0.001 | 0.645 | 1.03 ± 0.23 | 1.04 ± 0.25 | 0.671 | 0.007 |
| Admission AST (U/L) | 35.0 ± 10.1 | 30.1 ± 8.3 | 0.009 | 0.103 | 34.1 ± 10.0 | 29.2 ± 9.9 | 0.091 | 0.056 |
| Admission ALT (U/L) | 28.2 ± 9.2 | 23.1 ± 6.9 | 0.008 | 0.137 | 26.0 ± 8.1 | 24.1 ± 7.2 | 0.146 | 0.045 |
| Antihypertensive treatment | 477 (57.8%) | 357 (53.8%) | 0.158 | 0.077 | 139 (55.8%) | 135 (54.2%) | 0.787 | 0.032 |
| Statin | 446 (54.1%) | 380 (57.3%) | 0.223 | 0.066 | 139 (55.8%) | 134 (53.8%) | 0.719 | 0.040 |
| Anticoagulant | 109 (13.2%) | 150 (22.6%) | <0.001 | 0.267 | 41 (16.5%) | 45 (18.1%) | 0.952 | 0.044 |
| History of smoking | 312 (37.8%) | 224 (33.8%) | 0.149 | 0.105 | 92 (36.9%) | 85 (34.1%) | 0.700 | 0.076 |
| Hypertension | 612 (74.2%) | 527 (79.5%) | 0.019 | 0.127 | 186 (74.7%) | 181 (72.7%) | 0.684 | 0.046 |
| Dyslipidemia | 506 (61.3%) | 425 (64.1%) | 0.263 | 0.061 | 152 (61.0%) | 147 (59.0%) | 0.714 | 0.041 |
| Moderate-severe CKD | 39 (4.7%) | 192 (29.0%) | <0.001 | 0.686 | 15 (6.0%) | 11 (4.4%) | 0.546 | 0.072 |
| Atrial fibrillation | 114 (13.8%) | 161 (24.3%) | <0.001 | 0.191 | 47 (18.9%) | 50 (20.1%) | 0.821 | 0.030 |
| Coronary artery disease | 147 (17.8%) | 130 (19.6%) | 0.127 | 0.091 | 47 (18.9%) | 48 (19.3%) | 0.990 | 0.013 |
| Heart failure | 115 (13.9%) | 141 (21.3%) | 0.008 | 0.243 | 46 (18.5%) | 48 (19.3%) | 0.719 | 0.067 |
| COPD | 88 (10.7%) | 74 (11.2%) | 0.827 | 0.016 | 27 (10.8%) | 30 (12.9%) | 0.778 | 0.038 |
| Stroke | 84 (10.2%) | 103 (15.6%) | 0.003 | 0.160 | 27 (10.8%) | 26 (10.4%) | 1.000 | 0.013 |
| Dementia | 104 (12.6%) | 119 (17.9%) | 0.005 | 0.149 | 26 (10.4%) | 32 (12.9%) | 0.485 | 0.075 |
| Moderate-severe functional dependence | 156 (18.9%) | 218 (32.9%) | <0.001 | 0.337 | 54 (21.7%) | 60 (24.1%) | 0.791 | 0.061 |
| Moderate-severe comorbidity | 712 (86.3%) | 596 (89.9%) | 0.129 | 0.086 | 219 (88.0%) | 217 (87.1%) | 0.892 | 0.024 |
| Disease severity  Moderate  Severe  Critical | 612 (74.2%)  206 (25.0%)  8 (0.8%) | 482 (72.7%)  175 (26.4%)  6 (0.9%) | 0.289 | 0.067 | 181 (72.7%)  65 (26.1%)  3 (1.2%) | 182 (73.1%)  64 (25.7%)  3 (1.2%) | 0.782 | 0.059 |

Data are shown as mean ± standard deviations, absolute values, and percentages. A significant imbalance in the group was considered if a standardized mean difference between baseline variables of greater than 10%. Values were considered to be statistically significant when p<0.05.

The degree of functional dependence was assessed using the Barthel Index. The presence of comorbidities was assessed using the Charlson Comorbidity Index.

ALT: alanine aminotransferase; AST: aspartate aminotransferase; BG: blood glucose; CKD: chronic kidney disease; COPD: chronic obstructive pulmonary disease; GLD: glucose-lowering drugs; mg/dL: milligram/deciliter; SMD: standardized mean difference; U/L: unit/liter
