# Supplementary material for: Mortality and other adverse outcomes in patients with type 2 diabetes mellitus admitted for COVID-19 in association with glucose-lowering drugs: a nationwide cohort study
Source: BMC Med. 2020 Nov 16;18:359. doi: 10.1186/s12916-020-01832-2 (PMC7666969; doi:10.1186/s12916-020-01832-2)
Supplement: Supplementary file 2 — Additional file 2: Table S2. Pre- and post-propensity score matching of baseline sociodemographic and clinical characteristics of patients with type 2 diabetes mellitus admitted for coronavirus disease 2019 treated with dipeptidyl peptidase-4 inhibitors versus other glucose-lowering drugs. [file 12916_2020_1832_MOESM2_ESM.docx]

|  | Pre-propensity matching | | | | Post-propensity matching | | | |
| --- | --- | --- | --- | --- | --- | --- | --- | --- |
|  | DPP-4i (n=180) | Other GLD (n=1409) | p-value | SMD | DPP-4i (n=105) | Other GLD (n=105) | p-value | SMD |
| Age (years) | 78.8 ± 7.1 | 74.7 ± 8.2 | <0.001 | 0.396 | 80.0 ± 5.6 | 79.4 ± 5.0 | 0.961 | 0.006 |
| Male gender | 107 (59.4%) | 886 (62.9%) | 0.395 | 0.073 | 63 (60.0%) | 60 (57.1%) | 0.779 | 0.058 |
| Body Mass Index ≥30 | 55 (30.6%) | 396 (28.1%) | 0.810 | 0.027 | 31 (29.5%) | 38 (36.2%) | 0.378 | 0.142 |
| Admission BG (mg/dL) | 162.0 ± 39.4 | 151.0 ± 35.1 | 0.338 | 0.012 | 165.1 ± 40.1 | 153.4 ± 36.9 | 0.732 | 0.099 |
| Admission serum creatinine (md/dL) | 1.38 ± 0.31 | 1.02 ± 0.22 | <0.001 | 0.403 | 1.36 ± 0.30 | 1.19 ± 0.26 | 0.062 | 0.137 |
| Admission AST (U/L) | 30.5 ± 9.2 | 33.0 ± 10.1 | 0.089 | 0.114 | 29.0 ± 10.2 | 29.0 ± 9.9 | 0.507 | 0.080 |
| Admission ALT (U/L) | 23.0 ± 7.0 | 26.1 ± 7.9 | 0.036 | 0.145 | 23.0 ± 7.1 | 22.0 ± 7.0 | 0.531 | 0.044 |
| Antihypertensive treatment | 100 (55.6%) | 796 (56.5%) | 0.841 | 0.022 | 54 (51.4%) | 58 (55.2%) | 0.678 | 0.076 |
| Statin | 110 (61.1%) | 783 (55.8%) | 0.200 | 0.109 | 65 (61.9%) | 68 (64.8%) | 0.775 | 0.059 |
| Anticoagulant | 47 (26.1%) | 215 (15.3%) | 0.003 | 0.276 | 27 (25.7%) | 36 (34.3%) | 0.630 | 0.191 |
| History of smoking | 65 (36.1%) | 498 (35.3%) | 0.881 | 0.041 | 38 (36.2%) | 35 (33.3%) | 0.481 | 0.168 |
| Hypertension | 153 (85.0%) | 1062 (75.4%) | 0.006 | 0.242 | 87 (82.9%) | 91 (86.7%) | 0.565 | 0.106 |
| Dyslipidemia | 116 (64.4%) | 890 (63.2%) | 0.818 | 0.025 | 68 (64.8%) | 67 (63.8%) | 1.000 | 0.020 |
| Moderate-severe CKD | 58 (32.2%) | 167 (11.9%) | <0.001 | 0.510 | 30 (28.6%) | 27 (25.7%) | 0.756 | 0.064 |
| Atrial fibrillation | 51 (28.3%) | 230 (16.3%) | <0.001 | 0.291 | 27 (25.7%) | 36 (34.3%) | 0.228 | 0.051 |
| Coronary artery disease | 40 (22.2%) | 267 (18.9%) | 0.081 | 0.198 | 20 (19.0%) | 19 (18.1%) | 0.852 | 0.063 |
| Heart failure | 45 (25.0%) | 173 (12.3%) | <0.001 | 0.330 | 18 (17.1%) | 21 (20.0%) | 0.723 | 0.074 |
| COPD | 29 (16.1%) | 140 (9.9%) | 0.016 | 0.185 | 18 (17.1%) | 21 (20.0%) | 0.723 | 0.074 |
| Stroke | 24 (13.3%) | 171 (12.1%) | 0.721 | 0.037 | 10 (9.5%) | 11 (10.5%) | 1.000 | 0.032 |
| Dementia | 33 (18.3%) | 196 (13.9%) | 0.139 | 0.120 | 19 (18.1%) | 19 (18.91%) | 1.000 | 0.001 |
| Moderate-severe functional dependence | 60 (33.3%) | 327 (23.2%) | 0.010 | 0.232 | 32 (30.5%) | 37 (35.2%) | 0.444 | 0.176 |
| Moderate-severe comorbidity | 166 (92.2%) | 1216 (86.3%) | 0.006 | 0.276 | 100 (95.2%) | 102 (97.1%) | 0.721 | 0.099 |
| Disease severity  Moderate  Severe  Critical | 126 (70.0%)  448 (26.7%)  6 (3.3%) | 1042 (74.0%)  352 (25.0%)  14 (1.0%) | 0.098 | 0.101 | 75 (72.3%)  27 (25.7%)  3 (2.9%) | 77 (73.3%)  25 (23.8%)  3 (2.9%) | 0.865 | 0.048 |

Additional file 2: Table S2. Pre- and post-propensity score matching of baseline sociodemographic and clinical characteristics of patients with type 2 diabetes mellitus admitted for coronavirus disease 2019 treated with dipeptidyl peptidase-4 inhibitors versus other glucose-lowering drugs.

Data are shown as mean ± standard deviations, absolute values, and percentages. A significant imbalance in the group was considered if a standardized mean difference between baseline variables of greater than 10%. Values were considered to be statistically significant when p<0.05.

The degree of functional dependence was assessed using the Barthel Index.The presence of comorbidities was assessed using the Charlson Comorbidity Index.

ALT: alanine aminotransferase; AST: aspartate aminotransferase; BG: blood glucose; CKD: chronic kidney disease; COPD: chronic obstructive pulmonary disease; DPP-4i: dipeptidyl peptidase-4 inhibitors; GLD: glucose-lowering drugs; mg/dL: milligram/deciliter; SMD: standardized mean difference; U/L: unit/liter
