# Supplementary material for: Mortality and other adverse outcomes in patients with type 2 diabetes mellitus admitted for COVID-19 in association with glucose-lowering drugs: a nationwide cohort study
Source: BMC Med. 2020 Nov 16;18:359. doi: 10.1186/s12916-020-01832-2 (PMC7666969; doi:10.1186/s12916-020-01832-2)
Supplement: Supplementary file 3 — Additional file 3: Table S3. Pre- and post-propensity score matching of baseline sociodemographic and clinical characteristics of patients with type 2 diabetes mellitus admitted for coronavirus disease 2019 treated with insulin versus other glucose-lowering drugs. [file 12916_2020_1832_MOESM3_ESM.docx]

Additional file 3: Table S3. Pre- and post-propensity score matching of baseline sociodemographic and clinical characteristics of patients with type 2 diabetes mellitus admitted for coronavirus disease 2019 treated with insulin versus other glucose-lowering drugs.

|  | Pre-propensity matching | | | | Post-propensity matching | | | |
| --- | --- | --- | --- | --- | --- | --- | --- | --- |
|  | Insulin (n=292) | Other GLD (n=1458) | p-value | SMD | Insulin (n=129) | Other GLD (n=129) | p-value | SMD |
| Age (years) | 77.9 ± 9.0 | 75.0 ± 8.0 | 0.012 | 0.034 | 76.2 ± 8.5 | 74.4 ± 8.2 | 0.351 | 0.021 |
| Male gender | 161 (55.1%) | 939 (64.4%) | 0.003 | 0.193 | 75 (58.1%) | 85 (65.9%) | 0.248 | 0.160 |
| Body Mass Index ≥30 | 61 (20.9%) | 420 (28.8%) | 0.020 | 0.169 | 32 (24.8%) | 36 (27.9%) | 0.672 | 0.070 |
| Admission BG (mg/dL) | 178.2 ± 53.4 | 151.1 ± 40.8 | <0.001 | 0.398 | 171.2 ± 50.7 | 186.0 ± 46.9 | 0.655 | 0.041 |
| Admission serum creatinine (md/dL) | 1.35 ± 0.70 | 1.00 ± 0.20 | <0.001 | 0.597 | 1.22 ± 0.39 | 1.20 ± 0.36 | 0.934 | 0.069 |
| Admission AST (U/L) | 28.0 ± 8.5 | 33.0 ± 11.6 | 0.023 | 0.045 | 28.0 ± 8.2 | 29.0 ± 9.1 | 0.877 | 0.145 |
| Admission ALT (U/L) | 22.0 ± 7.7 | 26.0 ± 8.2 | 0.002 | 0.197 | 22.0 ± 6.2 | 22.0 ± 5.9 | 0.615 | 0.006 |
| Antihypertensive treatment | 143 (50.0%) | 843 (57.8%) | 0.011 | 0.169 | 69 (53.5%) | 67 (51.9%) | 0.901 | 0.031 |
| Statin | 146 (50.0%) | 849 (58.2%) | 0.015 | 0.160 | 64 (49.5%) | 62 (48.1%) | 0.901 | 0.031 |
| Anticoagulant | 61 (20.9%) | 219 (15.0%) | 0.002 | 0.218 | 22 (17.1%) | 22 (17.1%) | 1.000 | 0.001 |
| History of smoking | 81 (27.7%) | 552 (37.9%) | 0.004 | 0.225 | 37 (28.7%) | 49 (38.0%) | 0.279 | 0.199 |
| Hypertension | 224 (76.7%) | 1099 (75.4%) | 0.709 | 0.029 | 92 (71.3%) | 98 (76.0%) | 0.480 | 0.106 |
| Dyslipidemia | 176 (60.3%) | 934 (64.1%) | 0.307 | 0.070 | 77 (59.7%) | 70 (54.3%) | 0.451 | 0.110 |
| Moderate-severe CKD | 93 (31.8%) | 129 (8.8%) | <0.001 | 0.598 | 27 (20.9%) | 31 (24.0%) | 0.655 | 0.074 |
| Atrial fibrillation | 66 (22.6%) | 232 (15.9%) | 0.007 | 0.172 | 25 (19.4%) | 26 (20.2%) | 1.000 | 0.019 |
| Coronary artery disease | 58 (19.9%) | 292 (20.0%) | 0.472 | 0.052 | 25 (19.4%) | 26 (20.2%) | 0.822 | 0.041 |
| Heart failure | 53 (18.2%) | 277 (19.0%) | 0.102 | 0.088 | 22 (17.1%) | 24 (18.6%) | 0.911 | 0.029 |
| COPD | 25 (8.6%) | 160 (11.0%) | 0.261 | 0.082 | 11 (8.5%) | 15 (11.6%) | 0.535 | 0.103 |
| Stroke | 55 (18.5%) | 154 (10.6%) | <0.001 | 0.235 | 20 (15.5%) | 18 (14.0%) | 0.861 | 0.044 |
| Dementia | 62 (21.2%) | 181 (12.4%) | <0.001 | 0.237 | 23 (17.8%) | 21 (16.3%) | 0.869 | 0.041 |
| Moderate-severe functional dependence | 112 (38.4%) | 296 (20.3%) | <0.001 | 0.431 | 43 (33.3%) | 42 (32.6%) | 0.831 | 0.076 |
| Moderate-severe comorbidity | 261 (89.4%) | 1267 (86.9%) | 0.493 | 0.053 | 111 (86.0%) | 107 (82.9%) | 0.606 | 0.086 |
| Disease severity  Moderate  Severe  Critical | 205 (70.2%)  79 (27.1%)  8 (2.7%) | 1075 (73.7%)  366 (25.1%)  17 (1.2%) | 0.091 | 0.119 | 93 (72.1%)  33 (25.6%)  3 (2.3%) | 94 (72.9%)  34 (26.4%)  3 (2.3%) | 0.599 | 0.093 |

Data are shown as mean ± standard deviations, absolute values, and percentages. A significant imbalance in the group was considered if a standardized mean difference between baseline variables of greater than 10%. Values were considered to be statistically significant when p<0.05.

The degree of functional dependence was assessed using the Barthel Index. The presence of comorbidities was assessed using the Charlson Comorbidity Index.

ALT: alanine aminotransferase; AST: aspartate aminotransferase; BG: blood glucose; CKD: chronic kidney disease; COPD: chronic obstructive pulmonary disease; GLD: glucose-lowering drugs; mg/dL: milligram/deciliter; SMD: standardized mean difference; U/L: unit/liter
