# Supplementary material for: Mortality and other adverse outcomes in patients with type 2 diabetes mellitus admitted for COVID-19 in association with glucose-lowering drugs: a nationwide cohort study
Source: BMC Med. 2020 Nov 16;18:359. doi: 10.1186/s12916-020-01832-2 (PMC7666969; doi:10.1186/s12916-020-01832-2)
Supplement: Supplementary file 4 — Additional file 4: Table S4. Pre- and post-propensity score matching of baseline sociodemographic and clinical characteristics of patients with type 2 diabetes mellitus admitted for coronavirus disease 2019 treated with metformin plus dipeptidyl peptidase-4 inhibitors versus other glucose-lowering drugs. [file 12916_2020_1832_MOESM4_ESM.docx]

Additional file 4: Table S4. Pre- and post-propensity score matching of baseline sociodemographic and clinical characteristics of patients with type 2 diabetes mellitus admitted for coronavirus disease 2019 treated with metformin plus dipeptidyl peptidase-4 inhibitors versus other glucose-lowering drugs.

|  | Pre-propensity matching | | | | Post-propensity matching | | | |
| --- | --- | --- | --- | --- | --- | --- | --- | --- |
|  | Metformin + DPP-4i (n=288) | Other GLD (n=384) | p-value | SMD | Metformin + DPP-4i (n=127) | Other GLD (n=127) | p-value | SMD |
| Age (years) | 75.9 ± 7.1 | 76.8 ± 8.1 | 0.892 | 0.085 | 73.2 ± 7.5 | 76.7 ± 6.2 | 0.350 | 0.022 |
| Male gender | 186 (64.6%) | 211 (54.9%) | 0.013 | 0.202 | 76 (59.8%) | 75 (59.1%) | 1.000 | 0.016 |
| Body Mass Index ≥30 | 79 (27.4%) | 96 (25.0%) | 0.720 | 0.037 | 37 (29.1%) | 35 (27.6%) | 0.889 | 0.035 |
| Admission BG (mg/dL) | 169.7 ± 43.2 | 168.1 ± 50.2 | 0.674 | 0.191 | 167.2 ± 44.2 | 168.0 ± 49.9 | 0.954 | 0.031 |
| Admission serum creatinine (md/dL) | 1.00 ± 0.29 | 1.30 ± 0.39 | <0.001 | 0.563 | 1.02 ± 0.29 | 1.00 ± 0.35 | 0.182 | 0.041 |
| Admission AST (U/L) | 29.0 ± 9.5 | 29.0 ± 9.6 | 0.859 | 0.001 | 29.0 ± 9.2 | 28.0 ± 9.0 | 0.911 | 0.035 |
| Admission ALT (U/L) | 24.0 ± 8.1 | 23.0 ± 7.9 | 0.067 | 0.133 | 24.0 ± 8.0 | 24.0 ± 7.9 | 0.921 | 0.063 |
| Antihypertensive treatment | 167 (57.9%) | 196 (51.0%) | 0.098 | 0.139 | 71 (55.9%) | 70 (55.1%) | 1.000 | 0.016 |
| Statin | 183 (63.5%) | 200 (52.1%) | 0.005 | 0.228 | 73 (57.5%) | 67 (52.8%) | 0.528 | 0.095 |
| Anticoagulant | 42 (14.6%) | 80 (20.8%) | 0.082 | 0.210 | 15 (11.8%) | 19 (15.0%) | 0.761 | 0.093 |
| History of smoking | 108 (37.5%) | 116 (30.2%) | 0.157 | 0.153 | 47 (37.0%) | 43 (33.9%) | 0.864 | 0.068 |
| Hypertension | 215 (74.7%) | 295 (76.8%) | 0.630 | 0.045 | 91 (71.7%) | 89 (70.1%) | 0.890 | 0.035 |
| Dyslipidemia | 195 (67.7%) | 238 (62.0%) | 0.172 | 0.113 | 82 (64.6%) | 76 (59.8%) | 0.518 | 0.098 |
| Moderate-severe CKD | 22 (7.6%) | 109 (28.4%) | <0.001 | 0.561 | 12 (9.5%) | 14 (11.0%) | 0.836 | 0.052 |
| Atrial fibrillation | 43 (14.9%) | 87 (22.7%) | 0.015 | 0.200 | 16 (12.6%) | 19 (15.0%) | 0.716 | 0.069 |
| Coronary artery disease | 49 (17.0%) | 75 (19.5%) | 0.214 | 0.102 | 22 (17.3%) | 24 (18.9%) | 0.867 | 0.035 |
| Heart failure | 43 (14.9%) | 76 (19.8%) | 0.009 | 0.222 | 23 (18.1%) | 24 (18.9%) | 0.852 | 0.042 |
| COPD | 27 (9.4%) | 32 (8.3%) | 0.746 | 0.036 | 8 (6.3%) | 9 (7.1%) | 1.000 | 0.032 |
| Stroke | 36 (12.5%) | 64 (16.7%) | 0.164 | 0.118 | 13 (10.2%) | 15 (11.8%) | 0.841 | 0.050 |
| Dementia | 34 (11.8%) | 72 (18.8%) | 0.019 | 0.194 | 14 (11.0%) | 18 (14.2%) | 0.571 | 0.095 |
| Moderate-severe functional dependence | 61 (21.2%) | 128 (33.3%) | <0.001 | 0.299 | 27 (21.3%) | 31 (24.4%) | 0.794 | 0.085 |
| Moderate-severe comorbidity | 256 (88.9%) | 336 (87.5%) | 1.000 | 0.010 | 111 (87.4%) | 110 (86.6%) | 1.000 | 0.023 |
| Disease severity  Moderate  Severe  Critical | 206 (71.5%)  75 (26.0%)  7 (2.4%) | 277 (72.1%)  98 (25.5%)  9 (2.4%) | 0.564 | 0.077 | 91 (71.7%)  33 (26.0%)  3 (2.4%) | 91 (71.7%)  34 (26.8%)  2 (1.6%) | 0.718 | 0.088 |

Data are shown as mean ± standard deviations, absolute values, and percentages. A significant imbalance in the group was considered if a standardized mean difference between baseline variables of greater than 10%. Values were considered to be statistically significant when p<0.05.

The degree of functional dependence was assessed using the Barthel Index. The presence of comorbidities was assessed using the Charlson Comorbidity Index.

ALT: alanine aminotransferase; AST: aspartate aminotransferase; BG: blood glucose; CKD: chronic kidney disease; COPD: chronic obstructive pulmonary disease; DPP-4i: dipeptidyl peptidase-4 inhibitors; GLD: glucose-lowering drugs; mg/dL: milligram/deciliter; SMD: standardized mean difference; U/L: unit/liter
