# Supplementary material for: Mortality and other adverse outcomes in patients with type 2 diabetes mellitus admitted for COVID-19 in association with glucose-lowering drugs: a nationwide cohort study
Source: BMC Med. 2020 Nov 16;18:359. doi: 10.1186/s12916-020-01832-2 (PMC7666969; doi:10.1186/s12916-020-01832-2)
Supplement: Supplementary file 5 — Additional file 5: Table S5. Pre- and post-propensity score matching of baseline sociodemographic and clinical characteristics of patients with type 2 diabetes mellitus admitted for coronavirus disease 2019 treated with metformin plus sodium-glucose transporter 2 inhibitors versus other glucose-lowering drugs. [file 12916_2020_1832_MOESM5_ESM.docx]

Additional file 5: Table S5. Pre- and post-propensity score matching of baseline sociodemographic and clinical characteristics of patients with type 2 diabetes mellitus admitted for coronavirus disease 2019 treated with metformin plus sodium-glucose transporter 2 inhibitors versus other glucose-lowering drugs.

|  | Pre-propensity matching | | | | Post-propensity matching | | | |
| --- | --- | --- | --- | --- | --- | --- | --- | --- |
|  | Metformin + SGLT-2i (n=67) | Other GLD (n=583) | p-value | SMD | Metformin + SGLT-2i (n=34) | Other GLD (n=34) | p-value | SMD |
| Age (years) | 68.8 ± 4.0 | 78.3 ± 6.0 | <0.001 | 0.670 | 69.8 ± 4.2 | 73.8 ± 6.0 | 0.173 | 0.167 |
| Male gender | 46 (68.7%) | 331 (56.8%) | 0.083 | 0.248 | 22 (64.7%) | 21 (61.8%) | 1.000 | 0.061 |
| Body Mass Index ≥30 | 32 (47.8%) | 147 (25.2%) | <0.001 | 0.486 | 16 (47.1%) | 16 (47.1%) | 1.000 | 0.005 |
| Admission BG (mg/dL) | 141.1 ± 42.7 | 169.9 ± 61.5 | 0.004 | 0.367 | 146.0 ± 34.5 | 144.4 ± 39.4 | 0.976 | 0.056 |
| Admission serum creatinine (md/dL) | 0.89 ± 0.19 | 1.39 ± 0.48 | <0.001 | 0.648 | 0.82 ± 0.18 | 1.10 ± 0.36 | 0.168 | 0.032 |
| Admission AST (U/L) | 32.0 ± 7.5 | 30.0 ± 7.6 | 0.882 | 0.156 | 29.5 ± 7.2 | 29.0 ± 7.0 | 0.759 | 0.160 |
| Admission ALT (U/L) | 25.0 ± 7.1 | 23.0 ± 6.5 | 0.130 | 0.021 | 23.5 ± 6.0 | 22.0 ± 5.9 | 0.658 | 0.188 |
| Antihypertensive treatment | 40 (59.7%) | 306 (52.5%) | 0.342 | 0.140 | 71 (55.9%) | 70 (55.1%) | 1.000 | 0.016 |
| Statin | 49 (73.1%) | 329 (56.4%) | 0.014 | 0.351 | 25 (73.5%) | 26 (76.5%) | 1.000 | 0.068 |
| Anticoagulant | 7 (10.4%) | 140 (24.0%) | 0.059 | 0.420 | 3 (8.8%) | 4 (11.8%) | 1.000 | 0.144 |
| History of smoking | 24 (35.8%) | 190 (32.6%) | 0.596 | 0.158 | 13 (38.2%) | 10 (29.4%) | 0.560 | 0.283 |
| Hypertension | 47 (70.1%) | 469 (80.4%) | 0.065 | 0.244 | 24 (70.6%) | 24 (70.6%) | 1.000 | 0.001 |
| Dyslipidemia | 50 (74.6%) | 370 (63.5%) | 0.101 | 0.239 | 26 (76.5%) | 29 (85.3%) | 0.537 | 0.226 |
| Moderate-severe CKD | 3 (4.5%) | 184 (31.6%) | <0.001 | 0.755 | 2 (5.9%) | 1 (2.9%) | 1.000 | 0.144 |
| Atrial fibrillation | 9 (13.4%) | 149 (25.6%) | 0.040 | 0.312 | 3 (8.8%) | 4 (11.8%) | 1.000 | 0.097 |
| Coronary artery disease | 14 (20.9%) | 127 (21.8%) | 0.790 | 0.061 | 7 (20.6%) | 7 (20.6%) | 1.000 | 0.063 |
| Heart failure | 10 (14.9%) | 126 (21.6%) | 0.008 | 0.522 | 5 (14.7%) | 6 (16.2%) | 0.836 | 0.011 |
| COPD | 4 (6.0%) | 66 (11.3%) | 0.268 | 0.189 | 2 (5.9%) | 0 (0.0%) | 0.493 | 0.353 |
| Stroke | 6 (9.0%) | 99 (17.0%) | 0.128 | 0.241 | 3 (8.8%) | 4 (11.8%) | 1.000 | 0.097 |
| Dementia | 2 (3.0%) | 116 (19.9%) | 0.002 | 0.551 | 1 (2.9%) | 0 (0.0%) | 1.000 | 0.246 |
| Moderate-severe functional dependence | 8 (11.9%) | 207 (35.5%) | <0.001 | 0.602 | 3 (4.5%) | 5 (14.7%) | 0.751 | 0.189 |
| Moderate-severe comorbidity | 54 (80.6%) | 531 (91.1%) | 0.004 | 0.341 | 29 (85.3%) | 28 (82.4%) | 1.000 | 0.080 |
| Disease severity  Moderate  Severe  Critical | 47 (70.1%)  18 (26.9%)  2 (3.0%) | 420 (72.1%)  146 (25.0%)  17 (2.9%) | 0.402 | 0.099 | 24 (70.6%)  9 (26.5%)  1 (2.9%) | 24 (70.6%)  8 (23.5%)  2 (5.9%) | 0.412 | 0.092 |

Data are shown as mean ± standard deviations, absolute values, and percentages. A significant imbalance in the group was considered if a standardized mean difference between baseline variables of greater than 10%. Values were considered to be statistically significant when p<0.05.

The degree of functional dependence was assessed using the Barthel Index. The presence of comorbidities was assessed by the Charlson Comorbidity Index.

ALT: alanine aminotransferase; AST: aspartate aminotransferase; BG: blood glucose; CKD: chronic kidney disease; COPD: chronic obstructive pulmonary disease; GLD: glucose-lowering drugs; mg/dL: milligram/deciliter; SGLT-2i: sodium-glucose transporter 2 inhibitors; SMD: standardized mean difference; U/L: unit/liter
