# Supplementary material for: Mortality and other adverse outcomes in patients with type 2 diabetes mellitus admitted for COVID-19 in association with glucose-lowering drugs: a nationwide cohort study
Source: BMC Med. 2020 Nov 16;18:359. doi: 10.1186/s12916-020-01832-2 (PMC7666969; doi:10.1186/s12916-020-01832-2)
Supplement: Supplementary file 6 — Additional file 6: Table S6. Pre- and post-propensity score matching of baseline sociodemographic and clinical characteristics of patients with type 2 diabetes mellitus admitted for coronavirus disease 2019 treated with metformin plus insulin versus other glucose-lowering drugs. [file 12916_2020_1832_MOESM6_ESM.docx]

Additional file 6: Table S6. Pre- and post-propensity score matching of baseline sociodemographic and clinical characteristics of patients with type 2 diabetes mellitus admitted for coronavirus disease 2019 treated with metformin plus insulin versus other glucose-lowering drugs.

|  | Pre-propensity matching | | | | Post-propensity matching | | | |
| --- | --- | --- | --- | --- | --- | --- | --- | --- |
|  | Metformin + insulin (n=110) | Other GLD (n=1375) | p-value | SMD | Metformin + insulin (n=67) | Other GLD (n=67) | p-value | SMD |
| Age (years) | 74.6 ± 6.0 | 75.7 ± 6.9 | 0.720 | 0.049 | 74.1 ± 5.5 | 75.8 ± 6.9 | 0.572 | 0.137 |
| Male gender | 69 (62.7%) | 852 (62.0%) | 0.876 | 0.026 | 42 (62.7%) | 46 (68.7%) | 0.585 | 0.126 |
| Body Mass Index ≥30 | 39 (35.5%) | 361 (26.3%) | 0.038 | 0.222 | 29 (43.3%) | 29 (43.3%) | 1.000 | 0.002 |
| Admission BG (mg/dL) | 194.2 ± 54.5 | 150.5 ± 31.5 | <0.001 | 0.387 | 196.0 ± 64.1 | 155.1 ± 49.0 | 0.522 | 0.032 |
| Admission serum creatinine (md/dL) | 1.15 ± 0.29 | 1.07 ± 0.38 | 0.225 | 0.132 | 1.12 ± 0.28 | 1.03 ± 0.26 | 0.206 | 0.080 |
| Admission AST (U/L) | 35.0 ± 9.5 | 33.0 ± 8.6 | 0.294 | 0.056 | 36.5 ± 10.2 | 33.0 ± 8.6 | 0.437 | 0.112 |
| Admission ALT (U/L) | 24.0 ± 9.1 | 26.0 ± 10.5 | 0.489 | 0.013 | 26.1 ± 10.0 | 25.0 ± 9.9 | 0.6940 | 0.123 |
| Antihypertensive treatment | 67 (60.9%) | 766 (55.7%) | 0.355 | 0.102 | 42 (62.7%) | 42 (62.7%) | 1.000 | 0.001 |
| Statin | 73 (66.4%) | 750 (54.5%) | 0.017 | 0.253 | 46 (68.7%) | 53 (79.1%) | 0.238 | 0.240 |
| Anticoagulant | 16 (14.5%) | 228 (16.6%) | 0.302 | 0.164 | 8 (11.9%) | 7 (10.4%) | 0.923 | 0.174 |
| History of smoking | 36 (32.7%) | 493 (35.9%) | 0.562 | 0.109 | 22 (32.8%) | 19 (28.4%) | 0.768 | 0.126 |
| Hypertension | 90 (81.8%) | 1046 (76.1%) | 0.216 | 0.140 | 55 (82.1%) | 59 (88.1%) | 0.467 | 0.168 |
| Dyslipidemia | 82 (74.5%) | 853 (62.0%) | 0.013 | 0.269 | 52 (77.6%) | 51 (76.1%) | 1.000 | 0.035 |
| Moderate-severe CKD | 14 (12.7%) | 197 (14.3%) | 0.740 | 0.048 | 8 (11.9%) | 12 (17.9%) | 0.467 | 0.168 |
| Atrial fibrillation | 17 (15.5%) | 243 (17.7%) | 0.671 | 0.056 | 9 (13.4%) | 9 (13.4%) | 1.000 | 0.002 |
| Coronary artery disease | 27 (24.5%) | 321 (23.3%) | 0.189 | 0.132 | 16 (23.9%) | 16 (23.9%) | 1.000 | 0.038 |
| Heart failure | 19 (17.3%) | 183 (13.3%) | 0.311 | 0.109 | 11 (16.4%) | 10 (14.9%) | 1.000 | 0.042 |
| COPD | 12 (10.9%) | 150 (10.9%) | 1.000 | 0.002 | 5 (13.4%) | 6 (9.0%) | 1.000 | 0.054 |
| Stroke | 16 (14.5%) | 166 (12.1%) | 0.523 | 0.076 | 10 (14.9%) | 7 (10.4%) | 0.604 | 0.135 |
| Dementia | 17 (15.5%) | 206 (15.0%) | 1.000 | 0.013 | 9 (13.4%) | 7 (10.4%) | 0.790 | 0.092 |
| Moderate-severe functional dependence | 34 (30.9%) | 339 (24.7%) | 0.262 | 0.157 | 18 (26.9%) | 16 (23.9%) | 0.862 | 0.127 |
| Moderate-severe comorbidity | 95 (86.4%) | 1202 (87.4%) | 0.805 | 0.042 | 60 (89.6%) | 61 (91.0%) | 1.000 | 0.050 |
| Disease severity  Moderate  Severe  Critical | 69 (62.8%)  38 (34.5%)  3 (2.7%) | 960 (69.9%)  398 (28.9%)  17 (1.2%) | 0.090 | 0.113 | 44 (65.7%)  22 (32.8%)  1 (1.5%) | 46 (68.6%)  20 (29.9%)  1 (1.5%) | 0.799 | 0.093 |

Data are shown as mean ± standard deviations, absolute values, and percentages. A significant imbalance in the group was considered if a standardized mean difference between baseline variables of greater than 10%. Values were considered to be statistically significant when p<0.05.

The degree of functional dependence was assessed using the Barthel Index. The presence of comorbidities was assessed using the Charlson Comorbidity Index.

ALT: alanine aminotransferase; AST: aspartate aminotransferase; BG: blood glucose; CKD: chronic kidney disease; COPD: chronic obstructive pulmonary disease; GLD: glucose-lowering drugs; mg/dL: milligram/deciliter; SMD: standardized mean difference; U/L: unit/liter
